# Role of Mast Cells and Their Mediators in Chronic Kidney Diseases

**DOI:** 10.3390/ijms26209981

**Published:** 2025-10-14

**Authors:** Maria Tziastoudi, Christos Cholevas, Theodoros Eleftheriadis, Ioannis Stefanidis, Theoharis C. Theoharides

**Affiliations:** 1 Department of Nephrology, Faculty of Medicine, University of Thessaly, 41334 Larissa, Greece; matziast@med.uth.gr (M.T.); teleftheriadis@uth.gr (T.E.); stefanid@uth.gr (I.S.); 2 Laboratory of Pharmaceutical Technology, Division of Pharmaceutical Technology, School of Pharmacy, Faculty of Health Sciences, Aristotle University of Thessaloniki, 54124 Thessaloniki, Greece; ccholevas@auth.gr; 3 Institute for Neuro-Immune Medicine, Nova Southeastern University, Fort Lauderdale, FL 33314, USA; 4 Department of Immunology, Tufts University School of Medicine, Boston, MA 02111, USA

**Keywords:** fibrosis, flavonoids, histamine, inflammation, kidney, mast cell, mediators, proteases, tryptase

## Abstract

Chronic kidney disease (CKD) affects as many as 10% of the population, which translates to about 850 million globally. Even though genetics, diabetes, and hypertension contribute to CKD, the underlying pathologic processes remain poorly understood. Mast cells (MCs) are unique tissue immune cells capable of secreting numerous biologically active molecules. MCs have been associated with kidney diseases and poor CKD outcomes, but they have received limited attention in CKD research. MCs are typically located perivascularly and are identified through kidney biopsies, which limits their diagnostic utility. MC-specific biomarkers such as histamine and the proteases chymase and tryptase show potential, but signature biomarker profiles are needed. While MC biomarkers have been studied in non-renal diseases, their clinical relevance in kidney disease remains underexplored. This review aims to clarify the role of MCs in kidney diseases, such as diabetic nephropathy, IgA nephropathy, hypertensive nephropathy, pruritus, parathyroidism, renal amyloidosis, and lupus nephritis, as well as in conditions such as kidney fibrosis, inflammation, and kidney transplant rejection. Evidence indicates an increased number of MCs, as judged by increased urine levels of histamine, chymase, IL-33, metalloproteinase-9 (MMP-9), and tryptase. In conclusion, MCs are involved in the pathogenesis of CKD and may represent new targets for early diagnosis, prevention, and treatment.

## 1. Introduction

Chronic kidney disease (CKD) is a progressive condition marked by structural and functional kidney changes resulting from various causes [1]. It is typically defined by reduced kidney function, indicated by an estimated glomerular filtration rate (eGFR) below 60 mL/min per 1.73 m^2^, or by markers of kidney damage such as albuminuria, hematuria, or abnormalities detected through laboratory tests or imaging that persist for at least three months [2]. CKD is strongly associated with genetic predispositions [3,4] and with conditions like diabetes, glomerulonephritis, and cystic kidney diseases. However, the underlying causes of CKD are not yet fully understood. Moreover, nephropathy of unknown etiology remains without a clear pathogenesis. Nevertheless, immune-related gene polymorphisms have been reported [4], and most cases of CKD involve fibrosis and inflammation.

Mast cells (MCs) are unique tissue immune cells best known for their critical role in allergies, anaphylaxis, systemic mastocytosis (SM), and MC activation disorders (MCADs) [5,6,7] but also in autoimmunities [8] and inflammation [9,10,11,12]. MCs derive from bone marrow precursors and acquire different phenotypes depending on the local microenvironment. MCs contain as many as 1000 secretory granules per cell, express over 50 distinct surface receptors, and can store or newly synthesize as many as 350 biologically active mediators [13,14]. The best known surface receptors are those for stem cell factor (CSF); CD-117 (c-kit, a tyrosine kinase); the high-affinity FcεRI for immunoglobulin E (IgE), activated when crosslinked by antigen; the low-affinity Mass-related G-protein Coupled Receptor Member X2 (MRGPRX2), activated by cationic drugs and peptides; and toll-like receptors (TLRs), activated by pathogens [15,16,17,18]. As a result, MCs participate not only in allergic and anaphylactic reactions but also in inflammation [11,19,20,21].

Beyond their infiltration into the kidney, MCs also reside in surrounding connective tissues, where they become activated. Upon stimulation, they release numerous biologically active mediators (Table 1), including prestored granulemolecules, such as histamine chymase, tryptase, and TNF-α, as well as newly synthesized prostaglandins, leukotrienes, chemokines, cytokines, and growth factors [22]. These mediators contribute to inflammation, immune regulation, tissue repair, and remodeling in kidney disease [23,24]. There is a strong correlation between atopic diseases that depend on MCs and idiopathic nephrotic syndrome [25,26].

Although increased MC numbers and activation have been noted in various kidney diseases [27,28,29,30,31,32], limited research has explored the link between MC mediators and the progression of CKD.

## 2. Characteristics of Mast Cells in Kidneys

In healthy kidneys, MCs are very few, with counts around 0.5–1 per mm^2^ in humans, and mainly are located in the renal interstitium or capsule but rarely in glomeruli or tubules [33]. In contrast, other tissues—like skin, gut, and lungs—have far higher baseline MC densities that contribute to barrier immunity and allergic response [34]. However, kidney MCs notably increase in number (>20/mm^2^) during renal injury or fibrosis, regardless of the underlying disease [35]. This has been observed across conditions such as diabetic nephropathy, IgA nephropathy, pyelonephritis, and transplant rejection. Their abundance in diseased kidneys correlates strongly with interstitial fibrosis and declining renal function [36].

In humans, kidney-infiltrating MCs exhibit heterogeneity, including MC_TC_, which express both tryptase and chymase, and mucosal MC_T_, which express only tryptase, and rarely MC_C,_ which express only chymase [37]. The ratio of these subtypes varies depending on the renal disease [33]. In rejected kidney transplants, an additional MC type, staining only for chymase, has been identified [38].

Distribution patterns have not been studied in kidneys [39]. In one study of biopsy specimens 100 days after transplantation, it was reported that an increase in total MC numbers, along with a higher proportion of MC_TC_ among the total MC population in early biopsy specimens, was associated with progressive fibrosis and long-term graft function decline [40]. However, in disease states, kidneys may contain a mix of MC_T_, MC_TC_, and occasionally MC_C_, depending on the pathology and microenvironment [41].

The number of MCs within the kidneys is increased in kidney diseases, with their numbers sometimes rising 60-fold, whereas in the renal parenchyma of normal kidneys, MCs are sparse [33,42]. In both humans and mice, MCs are also present in the connective tissue of the kidney capsule, and they have been observed infiltrating draining lymph nodes, playing immunoregulatory roles [43,44].

A study of MCs isolated from human renal tumor tissue showed they express markers like c-kit (CD117), CD9, CD29, CD44, CD54, and CD63 but lack CD2, CD3, CD14, IL-3R, GM-CSFR, and C5aR, among others. MC also displayed tryptase positivity and minimal chymase, resembling MC_T_ phenotype [45].

Renal MCs contain and release key profibrotic mediators—tryptase, chymase, TGF-β1, and TNF—that stimulate fibroblast activation, collagen production, and inflammation [36]. In acute kidney injury (e.g., cisplatin-induced), MCs exacerbate damage via TNF-mediated neutrophil recruitment. Mast cell-deficient mice showed protection—confirming TNF as a key mast cell-derived mediator in acute injury [46] (Table 2).

## 3. Kidney Inflammation and Fibrosis

Mast cell numbers rise in diseases linked to chronic inflammation and have been correlated with the severity of interstitial fibrosis in patients with progressive CKD from various causes, as well as renal allograft dysfunction [28,29,34,36,47,48,49,50,51,52,53]. Interstitial fibrosis, a frequent feature of kidney disease, showed a positive correlation with the extent of MC infiltration [28,29,31,38,50]. Higher MC numbers were linked to poorer outcomes in kidney disease, whereas lower MC infiltration was seen in patients with stable or improving kidney function [27].

Furthermore, when human MCs were cultured together with fibroblasts, the interaction led to increased fibroblast proliferation, a process influenced by IL-4 through heterotypic cell-to-cell contact [54,55].

## 4. Renal Allograft Rejection

Kidney transplantation is the preferred treatment option for individuals with end-stage renal disease. However, organ rejection can occur often [56]. Mast cells are implicated in the development of both acute [57] and chronic allograft rejection [31]. In chronic allograft nephropathy, MCs were increased, alongside macrophages and T lymphocytes [57,58,59]. The number of chymase-positive MCs was reported to be increased and correlated with the severity of interstitial fibrosis in human renal allografts [38]. Another study demonstrated a strong correlation between MC_T_ and interstitial fibrosis in both grafted and non-grafted kidneys, suggesting that elevated MC numbers are not a phenomenon exclusive to transplanted kidneys [60]. A strong association was reported between the number of MCs present and the level of extracellular matrix buildup in kidney allografts [60]. These findings were independently confirmed [61,62].

In addition, immunofluorescence analysis showed that both MCs and activated basophils were present in cases of antibody-mediated rejection but were absent in control samples and those with interstitial fibrosis/tubular atrophy [63].

## 5. Mast Cell Presence in Kidney Diseases

### 5.1. Diabetic Nephropathy (DN)

It is well known that diabetic nephropathy is characterized by renal damage involving interstitial fibrosis and extracellular matrix buildup. A key feature is tubulointerstitial fibrosis, where studies reported a tenfold increase in MCs in the interstitium compared to healthy kidneys [32]. Another study also showed that MCs were present in large numbers in biopsies from patients with diabetic nephropathy [34].

ΜCs are believed to play a central role in initiating and sustaining fibrosis. Many MCs stain strongly for type VIII collagen, and their presence in periglomerular regions suggests they may contribute to collagen deposition there. Overall, increased MCs in DN may influence fibroblast activity and promote extracellular matrix formation [32].

### 5.2. IgA Nephropathy

MC_T_ were more commonly detected in kidneys affected by IgA nephropathy (IgAN) but were rarely seen in normal kidney tissue. Patients in the high-tryptase group exhibited more severe clinical and pathological features [64]. Additionally, this group showed greater infiltration of interstitial macrophages and lymphocytes compared to the low-tryptase group. An increased density of MC_T_ was also linked to a poorer prognosis in IgAN patients [64].

### 5.3. Hypertensive Nephropathy

The number of renal MCs was five times higher in individuals with hypertension compared to normotensive controls. [49] These cells were predominantly located in the peritubular interstitial areas, with some observed near blood vessels, but none within the glomeruli. Most MCs expressed IgE receptors, tryptase, and chymase, indicating a mature and differentiated phenotype in the context of hypertensive nephropathy. These findings suggest that the accumulation of MCs in the kidney may contribute to the development of hypertensive nephropathy in humans [49]. MC-chymase can produce angiotensin II, a potent vasoconstrictor [41,65].

### 5.4. Other Kidney-Related Diseases

Approximately 40% of patients with end-stage renal disease (ESRD) experience pruritus [66]. Those patients experiencing pruritus exhibited a higher number of MCs in the skin, and most were degranulated [67,68]. MC numbers were also significantly elevated in bone biopsies from women with hyperparathyroidism [69]. In rats, mature MCs accumulated at bone turnover sites, particularly at the bone–marrow interface after parathyroid hormone (PTH) treatment, preceding the onset of osteitis fibrosa. Patients with ESRD have high levels of PTH [69,70,71,72]. A number of studies showed that PTH could stimulate MCs [70,71].

MCs also have a crucial role in renal amyloidosis. More specifically, it has been found that MCs are a key component of the overall inflammatory response and play a significant role in the development of interstitial fibrosis in renal amyloidosis [73].

Moreover, MCs also play a key role in lupus nephritis via two distinct pathways of end-organ damage: one mediated by MCs in organ-specific autoimmunity and another MC-independent pathway in systemic autoimmunity [74]. Renal interstitial MC numbers vary among the different classes of proliferative lupus nephritis [75,76].

## 6. Mast Cell Mediators

It is important to identify biomarkers of acute kidney injury [77]. Owens et al. (2020) sought to create a predictive model based on biomarkers reflecting CKD pathophysiology, kidney function, and associated comorbidities [78]. Specifically, they developed a model using serum creatinine, osteopontin, tryptase, urea, and eGFR through linear discriminant analysis, which achieved an accuracy of 84.3%—surpassing traditional indicators such as eGFR, serum creatinine, and albuminuria [78]. Below, we discuss additional potential biomarkers, especially those associated with MCs, in addition to some markers of tubular damage (Table 3).

### 6.1. Tryptase

**Figure 1:**
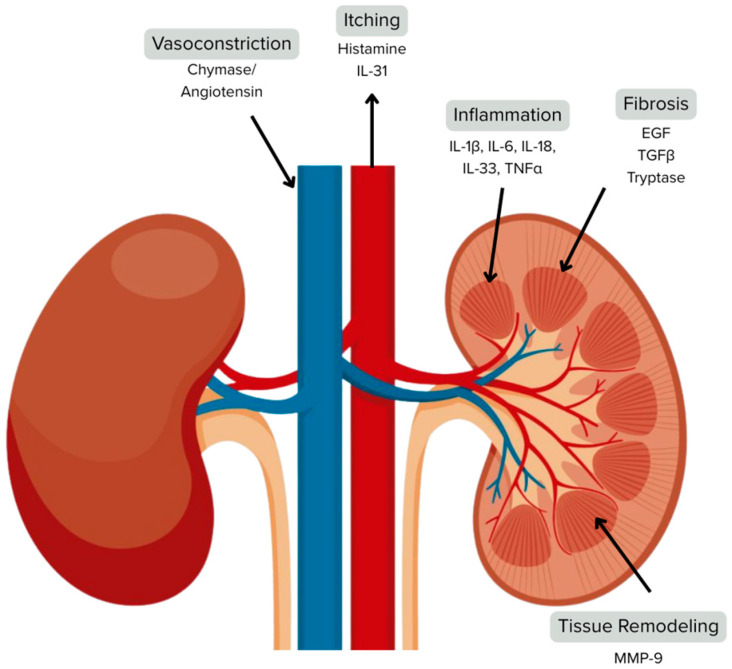
Schematic representation of the site of action and pathologic effect of select MC mediators.

Tryptase is a 30–35-kDa pro-inflammatory and profibrotic serine protease predominantly produced by MCs, with minor contributions from basophils [79,80,81]. Tryptase is stored in preformed secretory granules within MCs as a tetramer, inactivated by being bound to chondroitin sulfate (CS). Tryptase is secreted upon MC stimulation and is activated after dislodging from CS, contributing to various allergic and inflammatory responses [82]. There are multiple isoforms of tryptase, encoded by five loci on chromosome 16p, with α- and β-tryptase being the most clinically significant [83]. In healthy individuals, pro-tryptases are continuously secreted, whereas mature β-tryptase is secreted primarily during MC degranulation in events such as anaphylactic reactions and systemic mastocytosis (SM) [84]. Tryptase activates protease-activated receptor 2 (PAR2), which can stimulate tubular epithelial cells into a pro-inflammatory state [85]. Tryptase also activates protease-activated receptor 2 (PAR-2), which is widely expressed in various kidney cell types and plays a key role in inflammation and fibrosis in diseased kidneys [86,87,88,89,90,91]. PAR-2 activation in CKD is likely due to elevated MC infiltration [92]. Although present throughout the kidneys, expression of PAR-2 increases mainly in proximal tubular cells during kidney fibrosis, as seen in models like unilateral ureteral obstruction (UUO) and IgA nephropathy [47]. PAR-2 could contribute to disease progression by influencing TGF-β signaling, a central pathway in fibrosis [93]. Research has also indicated that tryptase plays a role in promoting fibrosis by inducing fibroblasts to produce type I collagen [94]. Additionally, tryptase has been reported to attract fibroblasts [95]. Tryptase has also been shown to act as a mitogen, promoting cell division in both epithelial cells—in which it stimulates IL-8 production and increases the expression of intercellular adhesion molecule-1—and microvascular endothelial cells, in which it contributes to angiogenesis and the formation of capillary-like structures [96,97] (Figure 1).

Normal serum tryptase levels range between 1 and 15 ng/mL, with an upper limit of approximately 11.4 ng/mL, though this threshold is debated among experts [79]. Its clinical utility is particularly evident when acute (within 4 h of an event) and baseline (at least 24 h after an event) serum levels are compared, following the consensus formula of a 20% + 2 ng/mL increase [98,99]. Recent publications increased the cut off limit to 20 ng/mL [100,101].

Elevated serum tryptase concentrations are observed in conditions such as systemic mastocytosis (>1 ng/mL), anaphylaxis, hypereosinophilic syndrome, many cases of mast cell activation syndrome (MCAS), and a subset of acute myelocytic leukemia [102]. It is noteworthy that while tryptase is detected in nasal secretions during allergic reactions, bronchoalveolar lavage fluid in asthmatic patients, and blood during anaphylaxis, it is generally not found in urine [103]. Tryptase is also elevated in hereditary alpha tryptasemia (HαT), an autosomal dominant genetic trait leading to excessive alpha tryptase production due to multiple *TPSAB1* copies, and affects about 6% of the population [104]. Interestingly, in one study of 105 patients with elevated serum tryptase levels, of those 57 with the highest tryptase levels, 37 had HαT, 7 had CKD, and 12 had myeloid disorders [105,106].

Ιncreased serum total tryptase (>11.4 microg/L) has been linked to decreased renal function and pruritus in hemodialysis patients [107]. Serum tryptase levels have been observed to rise as renal impairment progresses [108]. In the RIISC observational study of advanced and/or progressive CKD, higher baseline tryptase levels were linked to an increased risk of progression to ESRD in patients on ACEi/ARB therapy [48]. This association remained significant after adjusting for age, gender, eGFR, and ACR. While previous studies have shown tryptase levels rising with CKD stage, this is the first study to establish a connection between elevated serum tryptase and ESRD progression [48]. It has been found that tryptase stimulates the proliferation of renal fibroblasts and the production of collagen, especially when combined with heparin, another substance secreted by mast cells [28].

In CKD patients, serum tryptase levels were elevated—especially in advanced stages and in men—and negatively correlated with kidney damage indicators [108]. However, urinary detection of MC proteases is inconsistent and may depend on factors like sample concentration and collection method (e.g., spot vs. 24 h collection) [92].

### 6.2. Other MC Mediators

Chymase, another protease stored inside MC secretory granules, may have harmful effects by triggering inflammatory pathways, such as increasing nephrotoxic angiotensin II [109]. Beyond histamine and tryptase, a cluster of MC-linked mediators—IL-6, IL-9, IL-18, TGF-β, osteopontin, and VEGF—forms a tight feedback loop that worsens inflammation, fibrosis, and microvascular loss in CKD [15].

IL-9 and TNF-α were consistently elevated in the urine of acute interstitial nephritis (AIN) patients [110]. IL-6, released by both damaged tubular cells and infiltrating MCs, activates STAT3, boosts collagen I synthesis, and its rising plasma levels track with faster eGFR decline and poorer survival [92,111]. IL-6 also promotes MC proliferation [112]. IL-18, over-expressed in stressed tubules, primes MCs and—via its own STAT3 axis—worsens apoptosis, hypertension, and interstitial scarring, while circulating concentrations increase as renal clearance falls [113,114].

IL-31 is secreted from MCs [115] and has been associated with uremic pruritus [116,117,118]. The cytokine IL-33 has been implicated in allergic and inflammatory disorders [119,120] and has been shown to increase the release of cytokines and chemokines from cultured human MCs stimulated by the peptide substance P (SP) [121,122,123]. In fact, IL-33 has been implicated in kidney diseases [124,125,126].

Latent TGF-β, the master fibrotic switch, is enzymatically “unlocked” by MC tryptase and chymase, driving epithelial-to-mesenchymal transition and matrix expansion [39].

Osteopontin, a matricellular protein that both activates MCs and is secreted by them, is markedly up-regulated in CKD serum, urine, and biopsy tissue, where it recruits more inflammatory cells and stiffens the interstitium; blocking OPN expression in pre-clinical models attenuates renal scarring [127]. Osteopontin is secreted from MCs [128,129,130] and has been associated with kidney stones [131] and CKD [128,132], as well as arterial stiffness in patients on hemodialysis [133].

Monocyte chemoattractant protein 1 (MCP-1) recruits monocytes and MCs, thus amplifying the inflammatory process [134,135].

Additional molecules and some of their soluble receptors are listed in Table 2.

## 7. Interventions

There are no clinically effective drugs to prevent or inhibit CKD, especially kidney inflammation and fibrosis. Moreover, anti-inflammatory drugs are not commonly used due to the nephrotoxicity they exert [136,137]. The MC/CKD axis may be a reasonable treatment target. In rat models with CKD, drugs that can inhibit MCs (e.g., olopatadine, ketotifen, tranilast) reduced fibrosis, showing potential therapeutic benefit [138] (Table 4).

MC “stabilizers”, such as cromolyn sodium, have been reported to blunt renal scarring in animal models, while experimental chymase or tryptase inhibitors reduce fibrosis and preserve GFR more powerfully than renin-angiotensin blockade alone [80,139].

Certain naturally occurring flavonoids [140,141] appear to have important beneficial properties [142,143,144]. Luteolin and its structural analogue, tetramethoxyflavone, inhibit MCs in CKD [145,146,147,148,149,150]. Luteolin is more potent than cromolyn in its ability to inhibit mediator release from cultured human MCs [151,152]. Its MC -stabilizing capacity may constitute luteolin’s ability underlying the observation that dietary intake of luteolin is negatively associated with all-cause and cardiovascular mortality in chronic kidney disease patients [153]. Beyond epidemiology, mechanistic studies have has shown that luteolin mitigates renal ischemia-reperfusion injury via anti-inflammatory, anti-apoptotic, and Nrf2/HO-1-mediated antioxidant effects, reinforcing luteolin’s direct renal benefits [154]. The structurally related quercetin also inhibits MC degranulation, down-regulates NF-κB, and switches off the TGF-β/Smad signaling cascade that drives epithelial-to-mesenchymal transition; in diabetic, obstructive, and ischemic models, quercetin decreases collagen build-up and improves eGFR upward [155,156,157,158,159,160]. Luteolin and the similar flavonoid fisetin show similar dual action, decreasing MC cytokine release while dialing back SIRT1- or SMAD3-mediated fibroblast activation, and each reversed tubulointerstitial fibrosis in pre-clinical studies [161].

Other flavonoids, such as the green-tea catechin EGCG, best known as an antioxidant, directly inhibit MC calcium influx and mediator release while attenuating oxidative stress in the kidney [162]. The flavone glycoside, baicalin, was shown to reduce tubular damage and collagen deposition [163]. Flavonoid supplementation in CKD patients also shows promise in improving blood pressure, arterial stiffness, and oxidative stress markers [164]. These plant-derived compounds are orally bioavailable, generally safe, and—unlike biologic cytokine blockers such as tocilizumab (IL-6) or tadekinig-α (IL-18BP)—they tackle MCs, inflammation, oxidative stress, and fibrosis in one inexpensive package [165,166,167,168,169,170].

Surprisingly, recent papers indicated that the use of the vitamin niacin could reduce the risk of CKD and was inversely related to all-cause mortality in CKD patients [171,172,173,174,175]. One of the limiting factors of administering high amounts (>500 mg/day) of niacin is the associated “flush”, which is due to niacin-induced release of PGD_2_ from MCs [176]. However, it is interesting that the flavonoids luteolin and quercetin (as present in the liposomal formulationFibroPtotek^®^) could reduce the niacin-associated flush [177,178], in addition to providing the beneficial actions described above.

**Table 4:** Animal models of various kidney diseases where MC are implicated.

Kidney Disease/Model	Animal Model (Species/Induction Method)	Identified Role of Mast Cells	Main Findings	Limitations	References
Cisplatin-induced acute kidney injury	Mouse (C57BL/6); cisplatin administration	MC-derived TNF-α promotes neutrophil recruitment and tubular necrosis	MC-deficient mice protected from injury; confirms TNF as key MC mediator	Reflects acute toxicity, not chronic fibrosis	[46]
Crescentic glomerulonephritis	Male WBB6/F_1_-Kit^W^/Kit^Wv^ (W/W^v^) mice; Sheep anti-mouse-glomerular besement membrane (GBM) globulin serum injection	MCs amplify intrarenal chemokine production and adhesion molecule expression	MCs exacerbate glomerulonephritis, an association between mast cell numbers and interstitial inflammation was found	Short disease course; not fully representative of human GN	[179]
Experimental anti-GBM glomerulonephritis (W/Wv MC-deficient mice)	MC-deficient Kit^W^/Kit^W-v^ mice, MC-reconstituted Kit^W^/Kit^W-v^ mice, Kit^+/+^ control mice; immune complex–mediated glomerulonephritis	MCs exert protective effects by their ability to mediate remodeling and repair functions in nephrotoxic nephritis	Absence of MCs leads to dramatically increased glomerular damage	Model lacks full immune cell complexity	[44,180]
Ischemia–reperfusion injury (IRI)	Male Sprague Dawley (SD) rats; intraperitoneal injection of 3% pentobarbital sodium solution (30 mg/kg)	MCs activation increases inflammation and apoptosis	MCs stabilizers (luteolin, quercetin) reduce oxidative stress and fibrosis	Short-term model; no chronic progression	[154]
Lupus nephritis	Female mast cell-deficient WBB6F1/J-Kit^W^/Kit^W-v^ (W/Wv) and control congenic WBB6F1/J (WT) mice, intraperitoneal administration of 0.5 mL of pristane or phosphate-buffered saline (PBS)	MCs contribute to organ-specific autoimmune inflammation	MCs are dispensable in the development of humoral autoimmunity	Complex systemic autoimmunity; multifactorial effects	[74]
CKD intervention models (MC inhibition)	Rat or mouse models treated with MC stabilizers	MC inhibition reduces fibrosis and improves renal function	MC stabilizers and flavonoids suppress MC degranulation and TGF-β signaling	Preclinical; dosage and bioavailability differ across models	[138,139]

## 8. Limitations

Although MC accumulation in most nephropathies correlates with increased serum creatinine and fibrosis—suggesting a poor prognosis—some experimental studies suggest both disease-aggravating and protective roles [181,182].

Two studies using W/W^v^ mice showed that MCs may protect against glomerulonephritis by promoting tissue remodeling [44,180]. However, another study using the same mouse model found that MCs worsened disease by enhancing inflammatory TH1 responses [179]. Some data suggest chymase may have dual roles—either promoting or protecting against fibrosis—depending on the model and timing [183]. In addition, adhesion molecules are important in kidney development and function [184,185]. A major adhesion protein family is that of cadherins, which seem to be protective. E-cadherin is expressed by mast cells [186] and restricts MC activation [187]. However, many MC mediators, such as tryptase, destroy cadherins and minimize any beneficial action. The absolute number of MCs may vary depending on the stain (toluidine blue, c-KIT, tryptase) that was used and how the number of MCs was expressed (No/mm^2^ or No/HPF) [188]. In conclusion, however, it is the MCs’ activation that is more important than numbers [189].

## 9. Conclusions

Emerging evidence supports the involvement of MCs in CKD. Measurement of a panel of key MC mediators, along with biomarkers of kidney damage, may help with prognosis and early diagnosis. Moreover, inhibitors of MC activation may offer new treatment approaches. For instance, liposomal formulations in olive pomace oil to increase oral absorption of both luteolin and quercetin (FibroPtotek^®^) or only luteolin (PureLut^®^) are commercially available, have been used in several allergic and inflammatory conditions, and could be tested in pilot clinical studies.

## Figures and Tables

**Table 1:** MC kidney-relevant mediators.

Mediator	Action
Histamine	Angiogenenic, vasodilatory, pruritogenic
IL-1β, IL-6, IL-8, IL-33	Pro-inflammatory
MMP-9	Tissue disrupting
TNF-α	Pro-inflammatory
TGFβ	Profibrotic
Tryptase	Pro-inflammatory

**Table 2:** Characteristics of MCs in healthy versus diseased kidneys.

Feature	Kidney (Healthy) Nos/Levels/Site	Kidney (Diseased) Nos/Levels/Site
Mast Cell Density	Very low (<10 MC/HPF)	Substantially increased with fibrosis/injury
Localization	Interstitium/capsule; rare in glomeruli	Interstitium, perivascular, peri-tubular regions
Subtype Composition	Mostly MC_T_, some MC_TC_	Mixed MC_T_, MC_TC_, sometimes MC_C_
Functional Mediators	Tryptase (some), chymase (rare)	Increased Tryptase, chymase, TGF-β1, TNF
Role in Pathology	Sparse involvement	Significant in fibrosis, scarring, acute injury

HPF: high-power field.

**Table 3:** Mast cell mediators affecting kidney function.

Mediator	Pathologic Effect
Alpha-1 Macroglobulin (a1M)	Proximal tubule damage
Chymase	Angiotensin II production-vasocontriction
Cystatin C	Proximal tubule damage
Epidermal growth factor (EGF)	Kidney function
Histamine	TGFbeta production, inflammation, pruritus
IL-1beta	Inflammation
IL-1 soluble receptor	Inflammation
IL-6	Inflammation, MC proliferation
IL-8 (CXCL8)	Glucocyte chemotaxis
IL-9	Acute tubular necrosis
IL-18	Chronic inflammation
IL-31	Pruritus
IL-33	Inflammation
IL-33 soluble receptor	Inflammation
Kidney injury molecule 1 (KIM-1)	Tubular damage
Lipocalin 2 (LCN2)	Tubular damage
Monocyte chemoattractant peotein-1 (MCP-1)	Monocyte and mast cell recruitment
Metalloproteinase-9 (MMP-9)	Inflammation, tissue damage
Osteopontin	Fibrosis
TGFbeta	Fibrosis
TNFalpha	Inflammation, acute tubular necrosis
Tryptase	PAR2 activation, inflammation

## Data Availability

No new data were created or analyzed in this study. Data sharing is not applicable.

## Abbreviations

a1M	Alpha-1 Macroglobulin
ACEi	Angiotensin-converting enzyme inhibitors
ACR	Albumin–creatinine ratio
ARB	Angiotensin II Receptor Blockers
ATN	Acute tubular necrosis
CKD	Chronic kidney disease
CSF	Stem cell factor
EGCG	Εpigallocatechin Galate
EGF	Epidermal growth factor
eGFR	Estimated glomerular filtration rate
ESRD	End-stage renal disease
HαT	Hereditary alpha tryptasemia
HO-1	Heme-oxygenase *1*
HPT	Hyperparathyroidism
IgAN	IgA nephropathy
IgE	Immunoglobulin E
IL-1	Interleukin 1
IL-1beta	Interleukin 1 beta
IL-6	Interleukin 6
IL-18	Interleukin 18
IL-33	Interleukin 33
KIM-1	Kidney injury molecule 1
LCN2	Lipocalin 2
MCs	Mast cells
MCADs	MC activation disorders
MCAS	Mast cell activation syndrome
MCP-1	Monocyte chemoattractant protein 1
MMP-9	Metalloproteinase-9
MRGPRX2	Mass-related G-protein Coupled Receptor Member X2
Nrf2	Nuclear factor erythroid 2-related factor 2
OPN	Osteopontin
PAR-2	Protease-activated receptor 2
SM	Systemic mastocytosis
SP	substance P
STAT3	Signal transducer and activator of transcription 3
TGF-β	Transforming growth factor beta
TH1	*T helper 1*
TLRs	Toll-like receptors
TNFalpha	Tumor necrosis factor alpha
*TPSAB1*	Tryptase alpha/beta 1
UUO	Unilateral ureteral obstruction
VEGF	Vascular endothelial growth factor
